# Estimating the child health equity potential of improved sanitation in Nepal

**DOI:** 10.1186/1471-2458-13-S3-S25

**Published:** 2013-09-17

**Authors:** Anjali Acharya, Li Liu, Qingfeng Li, Ingrid K Friberg

**Affiliations:** 1 Department of International Health, Bloomberg School of Public Health, Johns Hopkins University, Baltimore, MD, USA

## Abstract

**Background:**

Access to improved sanitation plays an important role in child health through its impact on diarrheal mortality and malnutrition. Inequities in sanitation coverage translate into health inequities across socio-economic groups. This paper presents the differential impact on child mortality and diarrheal incidence of expanding sanitation coverage across wealth quintiles in Nepal.

**Methods:**

We modeled three scale up coverage scenarios at the national level and at each of the 5 wealth quintiles for improved sanitation in Nepal in the Lives Saved Tool (LiST): equal for all quintiles, realistically pro-poor and ambitiously pro-poor.

**Results:**

The results show that equal improvement in sanitation coverage can save a total of 226 lives (10.7% of expected diarrhea deaths), while a realistically pro-poor program can save 451 child lives (20.5%) and the ambitiously pro-poor program can save 542 lives (24.6%).

**Conclusions:**

Pro-poor policies for expanding sanitation coverage have the ability to reduce population level health inequalities which can translate into reduced child diarrheal mortality.

## Introduction

The past few years have witnessed an increased attention to health equity analyses describing the distributional impact of interventions [1-4]. These studies have aimed to analyze the extent to which interventions reach and benefit disadvantaged groups, such as the poor, certain ethnicities, or otherwise vulnerable populations [2]. Poor children are consistently found to be more likely to be exposed to health risks, and they have less resistance to disease because of undernutrition and other hazards typical in poor communities [5]. Compounding these inequities is reduced access to preventive and curative interventions [5].

Furthermore, recent papers have demonstrated that successive interventions are applied to the same population sub-groups, while the children in other sub-groups of populations consistently miss out, leading to a trend towards increasing inequity in child survival [6,7]. Co-coverage analyses show an inequitable clustering of interventions at the level of the child raises the possibility that the introduction of new technologies might primarily benefit children who are already covered by existing interventions [8]. This “inverse equity” in many countries implies that children who are most likely to fall sick are least likely to receive child health interventions [8,9]. Inequity patterns within countries are also found to be remarkably persistent over time, with only gradual changes from top inequity (disproportionately smaller gap for the wealthiest) in countries with coverage gaps exceeding 40% [10].

There is a growing recognition of the importance of addressing the underlying determinants of health, and that much of the work to redress health inequities lies beyond the health sector [11-13]. According to the report by the Commission on the Social Determinants of Heath, "*Water-borne diseases are not caused by a lack of antibiotics but by dirty water*, *and by the political*, *social*, *and economic forces that fail to make clean water available to all…*" [14]. Evidence has also shown that contextual factors including environmental characteristics such as water supply and sanitation may confound the delivery of a health sector intervention and its potential health impact [8].

Given its critical role in child health, inequities in access to environmental services (e.g. sanitation) then translate into health inequities across socio-economic groups. However, very few studies have looked at how scaling up such interventions differentially impacts different socio-economic groups. A study of the impact of improved water and sanitation in Stockholm from 1878 to 1925 showed a decline in overall mortality and of diarrhea mortality and a leveling out of socioeconomic differences in child mortality due to diarrheal diseases [15]. Another paper used comparative risk assessment modeling to estimate the reduction in child mortality as a result of improving child nutrition and providing clean water, sanitation, and fuels [16]. A study in Cameroon showed that improved household (water, sanitation and cooking fuel) and community environment had positive effects on a child’s nutritional status [17].

More recent research has provided evidence of increasing inequities in child health in developing countries, even as coverage of related interventions is expanding. Investigations of co-coverage of interventions to address child mortality reveal an “inverse” equity – which states that any new intervention will be adopted or received first by the wealthier classes, leading to increased inequities, before it is received by the poor [18]. Environmental health (EH) interventions (such as sanitation coverage) are closely associated with socio-economic status.

According to the 2011 Millennium Development Goals Report, the world is far from meeting the sanitation target – with almost two thirds of the people who practice open sanitation residing in Southern Asia [19]. Rural populations are at a disadvantage when it comes to improved sanitation. Inequalities are clearly most stark in South Asia, where an urban resident is 2.2 times more likely to use an improved sanitation facility than a rural resident [19]. For three countries in South Asia, an analysis of trends over the period 1995-2008 shows that improvements in sanitation have disproportionately benefited the wealthy [19]. Sanitation coverage for the bottom two quintiles of households has barely increased, and four out of five people in the poorest 40 percent continue to practice open defecation [19].

Given this, governments in South Asian countries, like Nepal, need to invest in expanding sanitation coverage – especially in the poorest households. Over the last few decades, Nepal has made significant improvements in access to safe water. The use of an improved water source for drinking water increased nationally from 65 percent in 1996 to 80 percent in 2006 [20]. According to the 2006 Nepal Demographic and Health Survey (DHS), the greatest improvements in access to safe water occurred among the poorest: while in 1996, access to safe water among the poor was only about one-third of that in the wealthiest quintile, by 2006 access to safe water was equally distributed across quintiles [20]. However, improvements in sanitation considerably lag behind improvements made in increasing access to safe water [20]. In 1996, 16 percent of the population had access to improved sanitation, with no one in the 2 poorest quintiles using improved sanitation in 1996 or 2001[20]. By 2006, 36 percent used improved facilities [20].

Given the vast differential in sanitation coverage between the wealth quintiles, there is potential for health improvements by investing in pro-poor sanitation in countries such as Nepal. This paper attempts to demonstrate the differential impact on child mortality and diarrheal incidence of expanding sanitation coverage across wealth quintiles through the use of the LiST model. A recent paper found LiST to be a valuable tool in providing estimates for wealth subgroups within a population, allowing users to compare alternative program scenarios based on the extent to which they would differentially prevent child deaths among the poorest populations [21]. The paper found that the modeled estimates of mortality within wealth quintiles fell within the 95% confidence intervals of measured mortality (in the Bangladesh Demographic and Health Survey) for both neonatal and post-neonatal mortality [21]. Another paper used the LiST model to estimate the potential lives saved from scaling up a number of diarrhea interventions at the national level for 68 countries – demonstrating the potential for drastically reducing diarrheal deaths [22].

This paper focuses specifically on sanitation coverage and looks at the population disaggregated by wealth quintile. Nepal is a good example as it continues to have the poorest sanitation coverage in South Asia and stark inequalities in coverage across wealth quintiles [20]. In addition, Nepal’s recent verbal autopsy linked to the DHS made the mortality estimates by wealth quintile more precise. The aim of this paper is to explore the potential of select sanitation interventions to differentially impact child mortality and morbidity, specifically to investigate the differential impact by wealth quintile of expanding coverage of sanitation on child mortality and diarrheal incidence in children.

## Methods

To carry out the analyses, we used the Lives Saved Tool (LiST) – which is designed to enable international agencies and country planners to estimate the effect of increasing coverage of selected intervention combinations, such interventions for diarrhea, on mortality. LiST utilizes country-specific cause of death profiles and the effect of selected interventions on cause-specific mortality, and thus generates country-specific estimates of mortality reductions. This tool can project the future number or rate of child deaths, and can stratify that projection by cause of death and by specific child health intervention based upon changes in health intervention coverage. These projections then can be used to enhance knowledge of child survival options among policymakers and to build support for effective activities. LiST requires information about child health and nutritional status, deaths by cause, and coverage of child health interventions, in addition to assumptions concerning the efficacy of those interventions. Included among a larger group of health-sector interventions, are a selected number of environmental health interventions to address childhood diarrhea.

**Figure 1:**
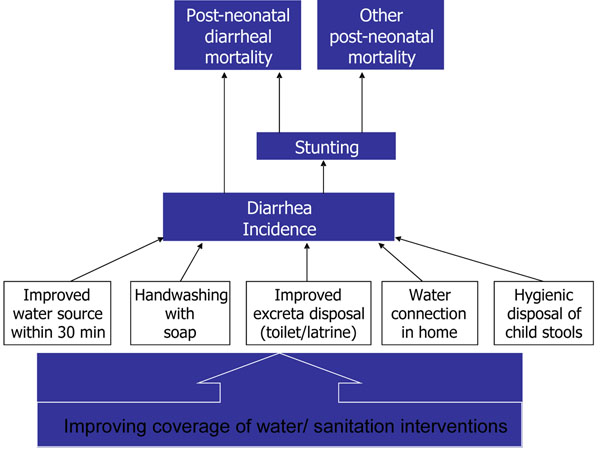
Conceptual framework for using LiST to estimate the lives saved from WSS interventions

All these interventions act through direct reduction of diarrhea deaths. However, they also reduce diarrheal incidence, which in turn has an effect on stunting, which has an effect on malaria, measles, diarrheal and pneumonia deaths. This paper is specifically focused on the issue of sanitation, and only the indicator relating to improved excreta disposal (sanitation) coverage for Nepal has been modeled. The conceptual framework is presented below. (Figure 1)

### LiST modeling

LiST supports program decision making by estimating the lives that can be saved by increasing coverage for proven maternal and child health interventions, alone or in combination, for user-defined populations and time frames. LiST generates estimates of deaths averted based on changes in coverage over time. In brief, LiST uses current health status (mortality rates, nutritional deficiencies and population sizes) in combination with current health intervention coverage values (i.e. ORS, facility delivery rates) to predict changes in mortality based on changes in health intervention coverage over time linked by effect sizes. LiST applies prevention effectiveness information prior to treatments, having each intervention impact only the residual number of deaths available to ‘‘save’’ for that year, thus eliminating the potential for double counting [23]. Within this general description, the targeted sanitation intervention (improved excreta disposal) has a direct impact on diarrheal mortality reduction, as well as an indirect impact on multiple causes of mortality via a reduction in the rate of stunting. LiST applies the documented effectiveness for each intervention to the total diarrheal deaths possible among children under 5 for each given year. Although there is uncertainty around both the inputs to LiST and the outputs from LiST, these are not considered in the current analysis.

### Establishing baseline values for cause of death and coverage of interventions

For this exercise, we generated LiST models for Nepal to project potential reductions in diarrhea mortality from changing sanitation coverage. At the national level, we used standard country-level child mortality rates from the 2006 Nepal DHS. For an estimate of baseline diarrheal deaths, the 2008 overall mortality rate and the cause of death structure were applied to the 2011 population. We also used baseline intervention coverage values for sanitation from the available Nepal DHS 2006 data. Using the available 2006 Nepal DHS survey data, health indicators (wasting, stunting) and intervention coverage (improved sanitation) were disaggregated by wealth quintile through the use of an asset index to divide the population.

Mortality rates by wealth quintile were available from the 2006 Nepal DHS. The data showed that infant mortality rate in the lowest quintile was 1.8 times higher, and under five mortality rate was 1.6 times higher than in the richest quintile.

The 2006 Nepal Demographic and Health Survey offered, for the first time in Nepal, a verbal autopsy survey that presents data on the proportional distribution of causes of death among neonates, post-neonates, and children age 12-59 months. Using this verbal autopsy data, the cause of death by wealth quintile for Nepal, for the post-neonatal age group (1-59 months) was recalculated. This data was then used as an input to the LiST model for cause of death in the 1-59 months age group; for the neonatal causes of death, default values were used [24]. Using standard methods for creating wealth quintiles, other health indicators such as the incidence of diarrhea, wasting and stunting, and population coverage by age groups were estimated using data from the Nepal 2006 DHS (See additional file 1).

In addition to health indicators, for the water and sanitation interventions covered in the LiST model, the relevant coverage data by wealth quintile for improved sanitary facilities, improved water within 30 minutes and piped water into household were calculated using standard methods and raw DHS data (Table 1). Piped water access in the richest quintile is over 40 times higher than in the poorest quintile; while sanitation coverage was nearly 18 times higher. Related hygiene behavior – reported handwashing practices and disposal of children’s stools – was also recorded to be significantly higher in the richest quintiles.

**Table 1:** Input data across wealth quintiles in Nepal

*Data*	*QUINTILE*					
Mortality Rates	poorest	poorer	middle	richer	richest	national

Neonatal	43.0	38.0	47.0	31.0	26.0	33.00
Infant	71.0	62.0	70.0	51.0	40.0	48.00
Under 5	98.0	83.0	91.0	63.0	47.0	61.00

Causes of Death (1-59 months)						

Diarrhea	11.9	14.63	19.51	13.04	13.64	14.22
Injuries	4.76	7.32	9.76	13.04	9.09	7.58
Measles	0	0	2.44	0	0	0.47
Other	45.24	51.22	29.27	39.13	36.36	41.71
Pneumonia	38.1	26.83	39.02	34.78	40.91	36.02

Diarrheal Incidence (<5 years)						

<1 month	0.00	0.00	0.00	0.00	2.61	0.51
1-5 months	3.02	2.37	3.41	3.72	4.27	3.30
6-11 months	3.56	2.34	3.14	3.16	2.15	2.95
12-23 months	2.00	1.55	1.59	1.53	1.75	1.71
24-59 months	16.48	15.35	10.54	9.29	12.65	13.31

Stunting (<-2 Z scores)						

< 1month	42.10	38.89	31.86	7.89	0.00	11.60
1-5 month	15.06	14.03	12.22	9.93	5.28	11.60
6-11 months	33.66	27.80	29.43	11.65	6.13	52.20
12-23 months	59.44	57.52	49.97	36.09	21.65	47.50
24-59 months	71.90	63.44	60.41	50.36	39.20	62.40
< 5 years	61.60	54.90	50.40	39.80	30.90	

Wasting (<-3 Z scores)						

< 1month	0.00	16.15	0.00	0.00	5.08	3.90
1-5 month	6.00	1.71	1.58	6.07	5.99	3.90
6-11 months	6.44	5.00	7.06	5.61	1.23	9.40
12-23 months	3.81	6.56	3.55	4.30	1.46	4.10
24-59 months	2.25	1.76	2.02	1.25	0.50	1.00
< 5 years	3.20	3.10	2.60	2.50	1.20	

Water, Sanitation and Hygiene						

Improved sanitary facility (%)	2.61	7.95	19.67	33.68	46.23	22.70
Improved water within 30 min (%)	85.2	87.7	84.3	87.7	79.5	79.8
Piped water into house/yard/plot (%)	0.91	4.34	7.45	12.88	36.58	*14.6*
Handwashing with soap (%)	37.7	52.3	59.7	77.9	87.9	64.1
Hygienic disposal of children's stools (%)	5.2	13.7	14.7	35.3	74.3	26.0

### Modeling increased coverage of interventions

The scale-up scenarios modeled assume a linear increase in coverage using the most recent data available, through the year 2015. This allowed for the estimation of the total number of diarrheal deaths and deaths averted in Nepal for each year between 2011 and 2015. Three alternative scale-up scenarios for sanitation, representing varying focus on expanding coverage across the different wealth quintiles in the Nepalese population, were applied:

(*a*) *Equal increase:* Using the MDG target for sanitation for Nepal (53% nationally), increasing improved sanitation coverage equally in each of the wealth quintiles to reach this target. Under this scenario, sanitation coverage for each quintile (q1-q4) was increased by roughly 2.6 times; while q5 was increased to the maximum of 99%. Altogether, the new sanitation coverage is 53 percent in 2015.

(*b*) *Pro-poor expansion* (*realistic*): Increasing improved sanitation coverage starting from the lowest quintile to the MDG national target (53%) in Nepal. Under this scenario, percentage increases in coverage were highest in q1, then in q2 and so on. To impart a degree of realism, increases in sanitation coverage were estimated such that for each quintile, the new coverage figure was lower than the new coverage in next highest quintile.

(*c*) *Pro-poor expansion* (*ambitious*): Simulating when sanitation programs are specifically targeted to poorest quintiles until they reach the MDG national target (53%) in Nepal. Under this, more ambitious pro-poor scenario, sanitation coverage in each wealth quintile was increased to reach the MDG target of 53 percent. The three scenarios are shown in Table 2.

**Table 2:** Alternative scenarios for expansion of sanitation in Nepal (% sanitation coverage)

QUINTILE	poorest	poorer	middle	richer	richest	Average
Current use of improved sanitary facility	2.6	8.0	19.7	33.7	46.2	22.7
Scenario (a): Equal increase	6.8	20.7	51.1	87.6	99.0	53
Scenario (b): Pro-poor expansion (realistic)	39.2	47.7	49.2	58.9	69.3	53
Scenario (c): Pro-poor expansion (ambitious)	53.0	53.0	53.0	53.0	53.0	53

## Results

From the three scenarios modeled using LiST, we were able to estimate the lives saved of children under five from diarrhea due to increases in sanitation coverage across various wealth quintiles (see Table 3). At the national level, if the sanitation coverage in Nepal were to reach the MDG target of 53 percent, it would result in averting approximately 529 deaths. This is approximately 24% of diarrheal deaths expected in Nepal if nothing were to change. Under the equal increase scenario, an estimated 236 lives (10.7%) would be saved by 2015. In the realistic pro-poor scenario, the LiST model estimated that 451 lives (20.5%) would be saved due to increased sanitation coverage; while the ambitious pro-poor scenario estimated 542 lives saved (24.6%).

**Table 3:** Deaths averted from alternate scenarios of expanding sanitation coverage

Scenarios ----->		a. Equal increase		b. Pro-poor (realistic)		c. Pro-poor (ambitious)	
Quintile	Current sanitation coverage%	New sanitation coverage%	Additional lives saved	New sanitation coverage%	Additional lives saved	New sanitation coverage%	Additional lives Saved

q1	**2.61**	**6.8**	26	**39.2**	208	**53.0**	285
q2	**7.95**	**20.7**	39	**47.7**	117	**53.0**	132
q3	**19.67**	**51.1**	91	**49.2**	85	**53.0**	96
q4	**33.68**	**87.6**	58	**58.9**	29	**53.0**	23
q5	**46.23**	**99.0**	22	**69.3**	12	**53.0**	6
Avg	22.7	53	**236**	53	**451**	53	**542**

### Diarrheal incidence

In addition to the direct impacts in terms of lives saved, the increase of sanitation coverage also impacts the incidence of diarrhea in children under five years of age. Under the ambitious pro-poor scenario, diarrheal incidence in the 24-59 age group declined by 18 percent in the lowest quintile (q1), by 17 percent in the second quintile (q2), and by 13 percent in the third quintile (q3), by 2015 over the baseline values in 2011 (Table 4).

Given the potential impacts of sanitation coverage on child health indicators, the LiST model was also used to ascertain the level of impact on stunting. However, results showed that even for the lowest quintile, stunting declined by less than half a percent between the baseline and ambitious pro-poor scenario in 2019 (data not shown).

**Table 4:** Decline in diarrheal incidence under different sanitation coverage scenarios

quintile	current sanitation coverage	Baseline	Scenario (a)	Scenario (b)	Scenario (c)	Baseline to Scenario (c) decline
q1	2.61	16.48	16.23	14.29	13.46	18%
q2	7.95	15.35	14.63	13.09	12.79	17%
q3	19.67	10.54	9.25	9.33	9.18	13%
q4	33.68	9.29	7.24	8.33	8.55	8%
q5	46.23	12.65	9.77	11.39	12.28	3%

## Discussion

In developing countries such as Nepal, sanitation coverage – an important contributor to child health – has been very much overlooked. Politically, attention to provide access to water, especially piped water, has received much more attention, and strategies to expand access to water have often focused on urban areas. This neglect of sanitation becomes even more stark when one looks at it through the lens of health equity – with lower socio-economic sections (as measured by wealth quintiles) of the population being disproportionately impacted. Knowing how health impacts from expanding sanitation coverage vary across wealth quintiles is critical input to government decision making.

This is the first application of the LiST model to the impacts of sanitation coverage on child health disaggregated across wealth quintiles, and can, and should, be replicated for other developing countries to inform policy dialog and contribute to government investment strategies. If sanitation coverage in Nepal were to reach the MDG target of 53 percent, it would result in averting approximately 529 deaths. This is about 24% of the approximately 2200 diarrheal deaths annually in children under five due to lack of adequate water and sanitation in Nepal [20,24]. However, this aggregate figure does not contribute to helping the government of Nepal in strategizing on which sub-populations to target by increased sanitation coverage. Through an analysis of alternative scenarios of sanitation expansion across the various quintiles, this analysis helps the Nepalese government with better targeting strategies. With currently available technology, the LiST model estimates that there could be as many as 542 fewer diarrheal deaths in Nepal by the end of 2015 if sanitation coverage was appropriately targeted to poorest households where environmental health conditions are the worst. This represents a 130 percent increase relative to the scenario of equal increase across wealth quintiles in the number of child lives saved from averted diarrheal mortality attributed to increased sanitation coverage – pointing to the potential for improving health equity by saving more lives amongst the poorest households in countries like Nepal.

Sanitation is particularly important for child health, not only in terms of the lives saved, but also because of the longer term impacts (such as IQ levels, school performance and future worker productivity) mediated through malnutrition [26,27]. Repeated diarrheal episodes contribute to malnutrition (stunting) in children under five – some of which is irreversible [28,29]. In this LiST application, results showed a considerable decline in diarrheal incidence especially under the pro-poor scenario when sanitation expansion was targeted to the lowest quintile – demonstrating the potential for lower rates of malnutrition and subsequent longer term health impacts.

These results suggest that LiST is a useful tool for policymakers to prioritize interventions by wealth quintile for maximal effect on diarrheal mortality and incidence in children under five years of age, at least in South Asia. However, the analysis in this paper also includes some weaknesses. For example, this analysis of health impacts of expanding sanitation coverage on different socioeconomic groups involves the use of asset indices to create wealth quintiles. There has been considerable debate about the composition of asset indices, and the use of principle component analysis to determine the weights. Index variables that were directly associated with child health outcomes (e.g. sanitation facility or source of water) increased inequality among households [30,31]. Therefore, there is some potential for some overestimation of inequality among the households in Nepal, as sanitation is included in the asset index in this analysis. In addition, this analysis only considers increased sanitation coverage, with other diarrhea-related interventions (both health and non-health) remaining constant in the period 2011-2015. Corresponding increases in other interventions such as access to improved water sources and piped water, handwashing practices, and increased ORS use would result in an even greater reduction in health inequities between the poorest and the richest subgroups in Nepal. Lastly, there is uncertainty associated with inputs and outputs of the LiST model, which has not been accounted for in this analysis.

Real progress can be made if the prevention and treatment of diarrhea becomes an international priority among governments in developing countries like Nepal. Increasing sanitation coverage in countries in South Asia, where sanitation lags far behind other environmental services, is critical, and requires inputs and leadership from, and coordination among, health, environment and infrastructure ministries. Coordinating especially the targeting of sanitation interventions to vulnerable population subgroups (such as the poorest quintile) is especially important to maxmize health benefits in terms of reduced child morbidity and mortality due to diarrhea.

The costing of the alternative scenarios was beyond the scope of this paper; but clearly resource considerations often constrain the rolling out of sanitation interventions in low income countries like Nepal. In a budget-constrained world, it becomes even more important to appropriately target these interventions to communities where the largest reductions in diarrheal mortality can take place, and to counter the tendency for co-coverage of many health and environmental interventions in richer households.

These results are generalizable to other developing countries with low levels of sanitation coverage which is unequal across wealth quintiles. While this analysis is disaggregated to the level of wealth quintiles, similar analyses can also be carried out at the provincial or district levels, based on the availability of further disaggregated data. This analysis is critical for program planners, funders, and policy and decision makers in developing countries like Nepal to better understand the potential impact on mortality when investing in diarrhea prevention at different wealth quintiles of the population.

Achieving the MDG national target of 53 percent sanitation coverage by 2015 is now practically out of reach for Nepal, and considerable opportunity has been lost for averting diarrheal deaths. However, looking ahead, the government and policy makers can take advantage of models such as LiST to build momentum towards expanding sanitation coverage, even while appropriately targeting non-health sector interventions such as improved water and sanitation hand-in-hand with other health sector interventions for addressing diarrhea (such as ORS use, vitamin A supplementation etc.). Working across sectoral ministries to improve health outcomes through interventions in both the health sector as well as other sectors will be critical in ensuring success in addressing child mortality in Nepal.

## Authors’ contributions

AA conceived the idea for the paper, performed the main analyses and was the lead author. IF provided technical guidance on the use of LiST, contributed to the writing, and provided overall quality control. QL reanalyzed the raw DHS data into wealth quintiles. LL recalculated data relating to cause of death by wealth quintile for Nepal, for the post-neonatal age group. All authors approved the final version of the paper.

## Competing interests

The authors declare no conflict of interests.

## Supplementary Material

Additional file 1 Methods and coverage data for Nepal Click here for file

## Declarations

The publication costs for this supplement were funded by a grant from the Bill & Melinda Gates Foundation to the US Fund for UNICEF (grant 43386 to "Promote evidence-based decision making in designing maternal, neonatal, and child health interventions in low- and middle-income countries”). The Supplement Editor is the principal investigator and lead in the development of the Lives Saved Tool (LiST), supported by grant 43386. He declares that he has no competing interests.

This article has been published as part of *BMC Public Health* Volume 13 Supplement 3, 2013: The Lives Saved Tool in 2013: new capabilities and applications. The full contents of the supplement are available online at http://www.biomedcentral.com/bmcpublichealth/supplements/13/S3.
