# Supplementary material for: Estimating the child health equity potential of improved sanitation in Nepal
Source: BMC Public Health. 2013 Sep 17;13(Suppl 3):S25. doi: 10.1186/1471-2458-13-S3-S25 (PMC3847615; doi:10.1186/1471-2458-13-S3-S25)
Supplement: Additional file 1 — Methods and coverage data for Nepal [file 1471-2458-13-S3-S25-S1.docx]

**Additional File: Definitions and Methods Used**

1. Population age structure: The Nepal 2006 DHS collected information from all usual residents of a selected household (the de jure population) and persons who had stayed in the selected household the night before the interview (the de facto population). In accordance with DHS convention and to maintain comparability and consistency with other indicators from DHS data, the age structure of de facto population was used in this analysis.

2. Stunting and wasting: The height-for-age index indicates linear growth retardation and cumulative growth deficits. Children whose height-for-age z-score is below minus two standard deviations (-2 SD) from the median of the reference population are considered short for their age (stunted) and chronically malnourished. Stunting reflects failure to receive adequate nutrition over a long period of time and is worsened by recurrent and chronic illness.

The weight-for-height index measures body mass in relation to body length and describes current nutritional status. Wasting represents failure to receive adequate nutrition in the period immediately preceding the survey and may be the result of inadequate food intake during a recent episode of illness, causing loss of weight and the onset of malnutrition. Children whose weight-for-height is below minus three standard deviations (-3 SD) from the median of the reference population are considered severely wasted. With age, height, and weight information from DHS data, the Z-score for each child under five years old was calculated using WHO Child Growth Standards.

3. Incidence of diarrhea. Incidence of diarrhea is defined as the number of cases divided by the total child-years lived. In Nepal 2006 DHS survey, interviewees were asked if their children under five years old they had diarrhea in the two weeks preceding the survey. As there were very few deaths in the preceding month (less than 1 percent) and there is no way to ascertain whether they died during the past two weeks. The analysis therefore ignored those deaths in calculating total child-years lived.

4. Use of improved sanitary facility: In this analysis, the definition of improved sanitary facility from Nepal 2006 DHS report was adopted. Only non-shared facility could potentially be considered as improved sanitary facility. Specifically, the following toilet types were included: flush - to piped sewer system; flush - to septic tank; flush - to pit latrine; pit latrine - ventilated improved pit; pit latrine - with slab; and composting toilet.

5**.** Creating Wealth Quintiles**:** One of the background characteristics used in the Nepal DHS 2006 report is an index of socioeconomic status. The economic index used in this study was developed and tested in a large number of countries in relation to inequalities in household income, use of health services and health outcomes (Rutstein et al., 2000). It is an indicator of the level of wealth that is consistent with expenditure and income measures (Rutstein, 1999). The economic index was constructed using household asset data including ownership of a number of consumer items ranging from a television to a bicycle or car, as well as dwelling characteristics, such as source of drinking water, sanitation facilities and type of material used for flooring.

Each asset is assigned a weight (factor score) generated through principal components analysis, and the resulting asset scores were standardized in relation to a normal distribution with a mean of zero and standard deviation of one (Gwatkin et al., 2000). Each household was then assigned a score for each asset and the scores were summed for each household; individuals were ranked according to the score of the household in which they resided. The sample was then divided into quintiles from one (lowest) to five (highest). A single asset index was developed for the whole sample; no separate indices were prepared for the urban and rural population. This classification of population by quintiles is used as a background variable in the following sections to assess the demographic and health outcomes in relation to socioeconomic status.

*Source: Nepal DHS 2006 Report*
